# Pelagic Life and Depth: Coastal Physical Features in West Africa Shape the Genetic Structure of the Bonga Shad, *Ethmalosa fimbriata*

**DOI:** 10.1371/journal.pone.0077483

**Published:** 2013-10-09

**Authors:** Jean-Dominique Durand, Bruno Guinand, Julian J. Dodson, Frédéric Lecomte

**Affiliations:** 1 Institut de recherche pour le développement, Laboratoire Ecologie des Systèmes Marins Côtiers UMR 5119, Université Montpellier II, Montpellier, France; 2 Institut des Sciences de l’Evolution de Montpellier UMR 5554, Université Montpellier II, Montpellier, France; 3 Département de biologie, Université Laval, Laval, Canada; 4 Direction de la faune aquatique, Direction de l’expertise sur la faune et ses habitats, Ministère du Développement Durable, de l’Environnement, de la Faune et des Parcs du Québec, Québec, Canada; University of Canterbury, New Zealand

## Abstract

The bonga shad, *Ethmalosa fimbriata*, is a West African pelagic species still abundant in most habitats of its distribution range and thought to be only recently affected by anthropogenic pressure (habitat destruction or fishing pressure). Its presence in a wide range of coastal habitats characterised by different hydrodynamic processes, represents a case study useful for evaluating the importance of physical structure of the west African shoreline on the genetic structure of a small pelagic species. To investigate this question, the genetic diversity of *E. fimbriata* was assessed at both regional and species range scales, using mitochondrial (mt) and nuclear DNA markers. Whereas only three panmictic units were identified with mtDNA at the large spatial scale, nuclear genetic markers (EPIC: exon-primed intron-crossing) indicated a more complex genetic pattern at the regional scale. In the northern-most section of shad’s distribution range, up to 4 distinct units were identified. Bayesian inference as well as spatial autocorrelation methods provided evidence that gene flow is impeded by the presence of deep-water areas near the coastline (restricting the width of the coastal shelf), such as the Cap Timiris and the Kayar canyons in Mauritania and Senegal, respectively. The added discriminatory power provided by the use of EPIC markers proved to be essential to detect the influence of more subtle, contemporary processes (e.g. gene flow, barriers, etc.) acting within the glacial refuges identified previously by mtDNA.

## Introduction

The spatial expansion of fisheries and its nefarious impact on marine ecosystems throughout the second half of the twentieth century is now well established [1]. From its development in northern and temperate waters, a southward expansion of fisheries occurred rapidly, with the greatest period of expansion occurring in the 1980s and early 1990s [1]. By the mid 1990s, two-thirds of all continental shelves were exploited [1]. This includes exploitation of marine ecosystems from the western coast of Africa, with marine fish landings increasing from 600,000 tonnes to approximately five million tonnes in the past sixty years [2]. Coastal African ecosystems have become the ‘fish basket’ of Europe and other countries in the last decades [3]. This increase in exploitation resulted in the degradation of ecosystems and the decline of biomass of target species that are often overexploited ( [4–8], but see 9). Projections on the sustainability of fish production in this area are pessimistic ([10,11]), because the synergistic action of exploitation, globalization and environmental stress occasioned by climate change may induce serious threats to the socioeconomic system of countries where fisheries are essential to develop sustainability (e.g. [12–15]).

Although numerous complementary techniques exist to define fish stocks [16], it is now well established that genetic data analyses are essential to better delineate stock structure for sustainable management (e.g. [17–19]). Exploited species from Western African ecosystems have been poorly studied using genetic approaches relative to their northern counterparts, thus impeding progress in stock delineation and fishery management. Indeed, as several coastal marine provinces and ecoregions are recognised along the western coast of Africa, such structure has most likely imprinted the genetic architecture ( [20,21]) thus increasing the potential for delineating stocks on a spatial basis. This spatial structure includes, from the north to the south: the Saharian upwelling in the Lusitanian province, the West Africa province (Mauritania, Senegal, The Gambia), the Gulf of Guinea province including countries from Guinea to Angola, and the Benguela province (Namibia, Western South Africa) [20]. However, studies relevant for genetic stock assessment have only concentrated at continental extremes. For example, Atarhouch et al. [22] demonstrated that a local Moroccan population of sardine (*Sardina pilchardus*) was genetically depleted as a probable consequence of intensive fishing in the recent past. Also considering sardine, Chlaida et al. [23] reported the likely existence of two populations in Moroccan waters and discussed the relevance of independent management of each stock. In the southern Atlantic, fishery genetics studies mostly concentrated around Cape Agulhas (e.g. [24–26]), and did not focus on the Atlantic slope of Africa from South Africa and Namibia to Angola. Population genetic studies dealing with species of economic importance along the rest of the western African coastline are rare, mostly using weakly polymorphic allozymes ([*Ethmalosa fimbriata*] [27]; [*Sarotherodon melanotheron*] [28,29]; [*Trachurus trecae*] [30]) or maternally inherited mitochondrial DNA (mtDNA) ([*Sardinella aurita*] [31]; [*E. fimbriata*] [32]). Only one such study focused explicitly on fishery management along the Angolan coast [30].

Previous studies dealing with the description of intra-specific phylogeographic relationships of marine coastal fish are generally congruent, indicating significant differentiation among populations of the Guinean province and populations located further north ( [27,28,31,32]). In the Bonga shad (*E. fimbriata*, Clupeidae), a euryhaline species distributed from Mauritania to Angola, Durand et al. [32] reported three well-defined phylogeographic units based on mtDNA: 1) a northern group extending from Mauritania to Guinea, 2) a central group distributed from Côte d’Ivoire to Cameroon, and 3) a southern group with populations extending from Gabon to Angola. However, allozymes and mtDNA might be of limited use to detect finer scale genetic structure [33], and increased exploitation of *E. fimbriata* demands further assessment of stock structure. This species is widely exploited in Western Africa with landings estimated to be ^~^225,000 tonnes ( [2], i.e., ^~^15% increase when compared to 2002). There is pressure to increase exploitation of this species, especially in Senegal where most landings occur and because of its importance in traditional fisheries [34]. This species is considered as having not yet suffered global depletion by overfishing; however, local depletion has been recently reported ( [35], for the Cross River, Nigeria). There is thus an opportunity to describe stock structure before heavy exploitation has the opportunity to obscure the natural order of connectivity.

**Figure 1:**
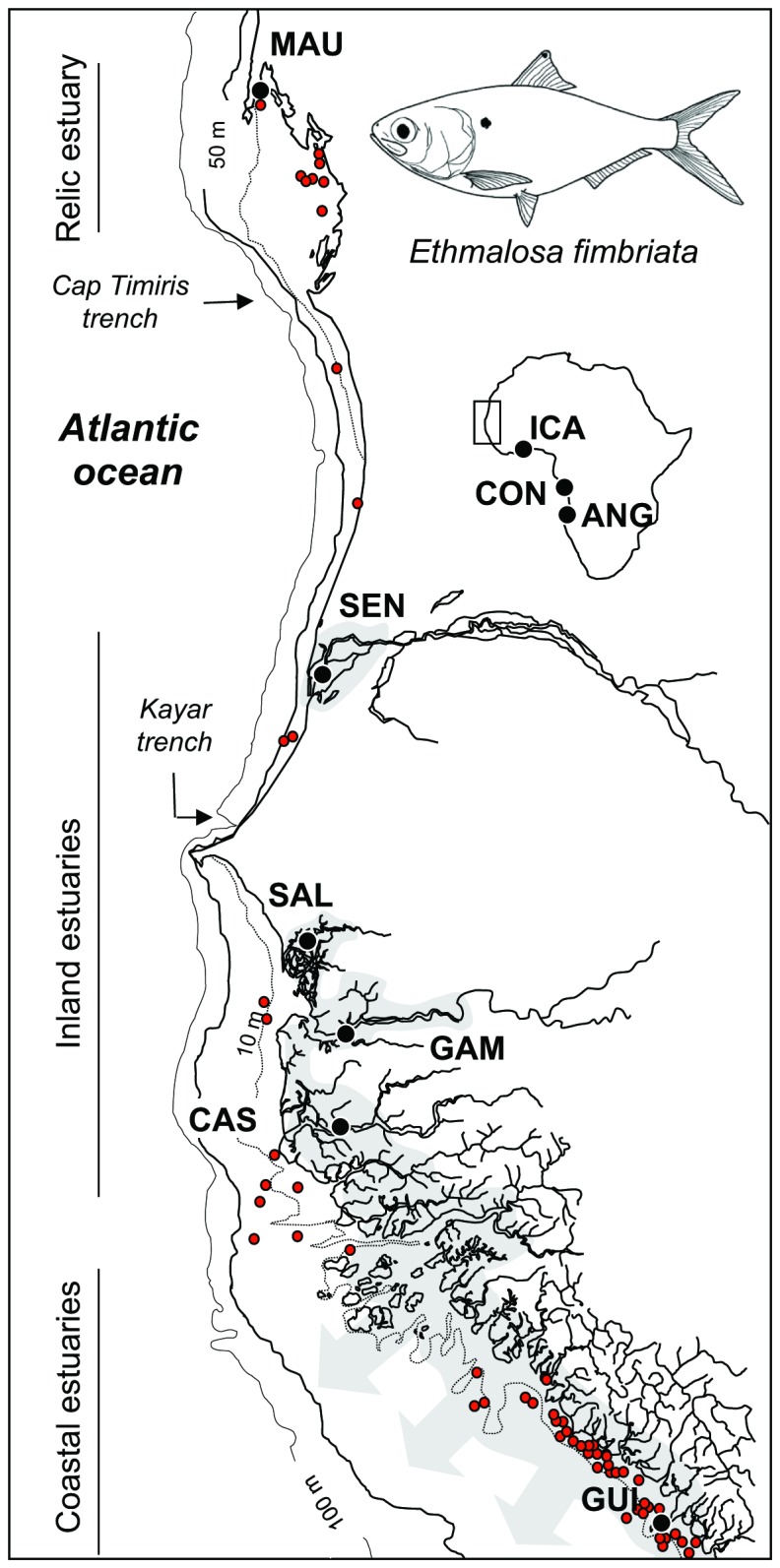
Sampling locations for Bonga shad. MAU: Etoile Bay, Mauritania; SEN: St Louis, Senegal; SAL : Foundiougne, Saloum, Senegal ; GAM : Tendaba, The Gambia ; CAS : Diogue, Casamance, Senegal ; GUI : Conakry, Guinea ; ICA : Aby, Côte d’Ivoire; CON: Loango Bay, Congo; ANG: Luanda, Angola. Grey areas represent estuarine conditions according to Charles-Dominique and Albaret [34]. Red dots indicate scientific sampling stations where Bonga shad were identified.

The distribution of *E. fimbriata* is partly patchy, being strongly dependent on the estuarine environment and river plumes that are mostly used for reproduction and as nurseries. Only larger individuals are found at sea [34]. Early tagging studies showed that dispersion of adults was restricted to shallow coastlines [36]. Avoidance of deep water was indicated by the absence of *E. fimbriata* from bottom and pelagic trawls where water is deeper that 10 m ( [37–39], Figure 1). 

In this context of exploitation, patchy distributions and the physical disruption of connectivity among populations, this study aims to define genetic variation and contemporary population structure in *E. fimbriata* and discuss possible mechanisms responsible for the observed structure. Using seven exon-primed intron-crossing (EPIC) markers, we first present results covering the distribution area of *E. fimbriata* and compare results to former allozyme and mtDNA data. We then more specifically focus on the northern range of the distribution (Guinea to Mauritania), the northern group of Durand et al. [32], as most landings are concentrated there.

## Materials and Methods

### Sampling area

The Bonga shad (*Ethmalosa fimbriata*) exploits coastal areas and a wide range of estuarine environments [34]. According to hydrodynamic features and climatic conditions, four types of estuarine environments are distinguished along the west coast of Africa (Table 1) [34]. The Bonga shad inhabits in all of them, including: (1) inland estuaries, characterized by flat river valleys, alternatively flooded by the intrusion of marine water during the dry season followed by freshwater flooding during the rainy season (outflow is usually limited), (2) coastal estuaries, characterized by a large and permanent freshwater outflow to the sea, (3) coastal lagoons, characterized by permanent estuarine conditions limited to the lagoon, and (4) relic estuaries, characterised by the absence of estuarine conditions despite the presence of typical estuarine species. The latter type is rare, located where large rivers were flowing in the recent past. Hence, in the Banc d’Arguin located in Mauritania, the Bonga shad occurs with other typical estuarine species including *Sarotherodon melanotheron* [40]. The mangrove swamp in the Banc d’Arguin is a relic of a previously humid period (the African Humid Period) when this area was a vast estuary resulting from the confluence of large rivers draining what is now the Sahara desert ( [41,42]). This typology of estuaries was taken into account during sampling, and all types were sampled for the present study (Table 1; Figure 1). Moreover, extreme environmental conditions are found is some estuaries of this region, such as hypersalinity (Saloum and Casamance estuaries) (e.g. [43–45]).

**Table 1:** Sampling locations (from North to South) and details about the environments, the sampling date and the samples size (N).

Code	Location	Country	Ecosystem	Estuarine	Date	N
				environment		
MAU	Etoile Bay	Mauritania	Sea	relic estuaries	Feb. 2002	61
SEN	St Louis, Senegal River	Senegal	Estuary	inland	Nov. 2001	61
SAL	Foundiougne, Saloum	Senegal	Estuary	inland	July 2001	48
GAM	Tendaba, Gambia River	The Gambia	Estuary	inland	May 2001	60
CAS	Diogue, Casamance	Senegal	Estuary	inland	Feb. 2002	60
GUI	Conakry	Guinea	Sea	coastal	Feb. 2002	55
ICA	Aby	Côte d’Ivoire	Lagoon	lagoon	July 2001	50
CON	Loango Bay	Congo	Sea	inland	Oct. 2001	54
ANG	Luanda	Angola	Sea	inland	July 2002	31

### Sampling

Adults of *E. fimbriata* were mostly collected between 2001 and 2002 in Mauritania, Senegal, the Gambia, and Guinea (Figure 1). The samples were collected from fish landing sites where no specific permissions were required. This study complied with all relevant regulations in countries where samples were obtained. Three additional samples were added to compare the level of molecular differentiation observed within this northern group to populations distributed over the entire natural range of Bonga shad (ICA: Côte d’Ivoire; CON: Democratic Republic of Congo; ANG: Angola; Figure 1). ICA belongs to the central mtDNA group, while CON and ANG belong to the southern mtDNA groups defined in Durand et al. [32]. A total of 480 fish were sampled (Table 1). Adults were all obtained from landings of artisanal fisheries, caught using castnets, purse seines or drift nets. A piece of pectoral fin was preserved in 95% ethanol.

### Molecular methods

Total DNA was extracted using the GenElute miniprep kit (Sigma-Aldrich). DNA was resuspended in 50 to 100 µl of ultrapure water. We considered the variability of seven nuclear loci using the EPIC PCR (exon-primed, intron-crossing polymerase chain reaction) method to assess the variation through electrophoresis (e.g. migratory pattern is used as a proxy to define allelic variants). Introns amplified in this study include the fourth intron of the aldolase B gene (*AldoB*); the second intron of two different glyceraldhyde-3-phosphate deshydrogenase genes (*Gapdh slow* and *Gapdh fast*, respectively); the sixth intron of three distinct creatine kinase (CK) isoforms (*CK1*, *CK2, CK3*) (details below), and an anonymous nuclear-DNA locus (*Myoglo2*). The *AldoB* and the two *Gapdh* loci were amplified using, respectively, primers *Aldo 5F*/*Aldo 3.1R* and *Gpd 2F*/*Gpd 3R* of Hassan et al. [46]. Locus *Myoglo2* was amplified using primers *Myo2EFI F* and *Myo2EFI R* of Panfili et al. [43]. The *CK1*, *CK2, CK3* loci were simultaneously amplified using primers *CK6F* and *CK7R* [47]. To discriminate the three *CK* loci, we excised and re-amplified the three bands obtained after migration of the initial amplicon containing the three loci on a 6% denaturing polyacrylamide gel. The product of the second amplification was purified using a PCR Product Pre-Sequencing kit (USB Corporation) and sequenced. We performed a BLAST search [48] in GenBank to identify which loci correspond to each isoform of *CK*. Sequences obtained were used to design specific reverse primers for each *CK* isoform. Primer *CK6F* was then used either with *CK6-1EFI R* (5’- TCCTGGATGAGATGCTCCAC-3’), *CK6-2EFI R* (5’-TCTCCTGAATCAGCCTCTCC-3’) or *CK6-3EFI R* (5’- TTGAATAAGGACTCAATCTG-3’) for amplification of the three *CK* loci.

Conditions for the amplification of the *AldoB*, *Myoglo2, Gapdh slow* and *Gapdh fast* loci are reported in Panfili et al. [43]. Amplification of the three *CK* loci was performed in 10 to 20 µl of reaction mixture, containing 1µl DNA template, 0.25U Taq polymerase in its buffer (Promega), 0.4µM of each primer, 1.5mM MgCl_2_, and 74µM each of dNTP. After one denaturing step during 3 min at 94°C, PCR was achieved in 35 cycles consisting of a 30s denaturation step at 94°C, a 30 s annealing step at 50°C and a final 30 s extension step at 72°C. A final extension step of 10 min at 72°C was added after the last cycle. The *CK6F* primer was labelled at the 5’ end with the fluorochrome 6-FAM (Sigma Genosys). Amplification products (10 µl) were mixed with 10 µl of formamide loading dye and denatured at 94°C for 5 min. Three microliters of denatured PCR products were loaded into 6% denaturing polyacrylamide gel, and run using 1X TBE buffer. Locus polymorphisms were screened at 505 nm (6-FAM) and visualized using a Hitachi FMBIO II scanner (Hitachi Instruments). Reference samples were used to standardize results.

### Statistical analysis

#### Intra-population genetic diversity

Observed heterozygosities (*H*_*obs*_) were estimated using the Genetix v.4.05 software [49]. Deviations from Hardy-Weinberg expectations (HWE) within each sample were investigated using *f* [50], an analogue to Wright’s fixation index, *F*_*is*_ [51]. The null hypothesis (f = 0) of no significant departure from panmixia was tested by randomly permuting alleles (*n* = 1000) from the original matrix of genotypes. The likely presence of null alleles was inspected using MicroChecker [52]. When null alleles were identified in a population, allelic frequencies were re-estimated using the Brookfield correction [53]. Linkage disequilibrium between pairs of nuclear loci (i.e. non-random associations of particular genotypes) was tested with the Genepop’007 software using exact tests [54]. Sequential Bonferroni correction was applied in multiple HWE and LD tests.

#### Genetic bottlenecks

The potential imprint of a reduction in effective population size (i.e. population bottleneck) on the nuclear diversity was tested using the M-ratio method described in Garza and Williamson [55]. This method does not suffer limitations when using a moderate number of loci. M represents the ratio of the number of alleles to the total range in allele size. Computations were run for all loci separately, and average M computed across loci in each population. Values of M depended on several parameters, such as the percentage of one-step mutations (*g* = 1, i.e., each mutation changes allele size by only one repeat unit), the size of alternate mutations that are not one-step (*g* ≥ 2; two-phase model [56]), and Θ defined as four times the product of effective population size (*N*_e_) and mutation rate (*μ*). Different relative combinations of one- and multiple-step mutations were investigated over a range of parameters as in Guinand and Scribner [57]. Results were not found to vary significantly (results not shown), and results reported in the present study are for a proportion of 85% one-step and 15% multiple-step mutations, *g* = 3, and Θ (10000 iterations). The value of Θ used to compute the M-ratio test was derived from the maximum-likelihood estimation described below. For each sample, a population at equilibrium was simulated 10 000 times for each combination of these parameters and simulated values of M (M_sim_) were computed. The empirical estimate of M across loci was then compared to the distribution of M_sim_ values to assess significance (95% criterion).

#### Population structure and patterns of genetic differentiation

Levels of population differentiation were estimated by *F*_ST_ [50], an estimator of F_ST_ [51], using Genetix v.4.05. Significance levels for all pairwise tests were corrected for multiple comparisons with the sequential Bonferroni procedure [58]. The population genetic structure was further investigated using two different methods aimed at detecting spatial population structure and locating discontinuities in allele frequencies. A clustering method implemented in the software TESS [59] was first used. TESS is a spatially explicit Bayesian admixture model implementing a MCMC algorithm that estimates individual ancestry proportions by incorporating spatial trends and autocorrelation in the prior distribution [60]. As an ‘admixture model’, TESS assumes that the data originate from the admixture of *K* putative parental populations. Admixture proportions were computed for each individual in the sample and stored in a matrix, **Q**, with elements (*q*_*ik*_; *i* = individuals, *k* = sample) representing the proportion of the individual’s genome that originates from the parental population *K*. To determine the most probable value of *K*, the maximum number of clusters, *K*_max_, was sequentially increased until the final inferred number of clusters, *K*, was less than *K*_max_. First the non-admixture model was used with a burn-in period of 20,000 cycles, and estimation was performed using 30,000 additional cycles. The maximal number of clusters was increased from *K*_max_ = 1 to 7. The conditional, auto-regressive, Gaussian model of admixture with a linear trend surface was used [61]. MCMC algorithms were run for a length of 50,000 sweeps with burn-in periods of 40,000 sweeps. For each data set and each model, we ran the algorithm 100 times, retained the 20 runs with the best discriminant information criteria [61], and averaged admixture estimates using the CLUMPP v.1.1 software [62]. In order to compare and to confirm outcomes of TESS, the BARRIER v.2.2 program was also used to identify the geographic areas associated with genetic discontinuities at nuclear loci [63]. Monmonier’s algorithm, implemented in the program, identifies boundaries associated with the highest genetic heterogeneity on a map where the samples are represented according to their geographical coordinates and are connected by Delaunay triangulation, with edges associated by genetic differentiation measures (*F*_ST_). As for TESS, 2 to 8 implicit boundaries were tested to estimate the reliability of the method. To quantify the potency of the various boundaries we used a bootstrapping procedure on the seven loci matrix to define the support for the different scenarios tested (e.g. the number of predefined boundaries). Individuals genotyped for ≤5 loci were discarded from analyses made with TESS and BARRIER.

To determine the influence of geographic distance on genetic differentiation, we tested also for isolation by distance (IBD) by investigating the correlation between a geographic distance matrix (i.e. minimum coastline distance between all pairs of locations) and a genetic distance matrix [i.e. *F*_ST_ /(1-F_ST_) distance; Rousset [64]]. The significance of the correlation (z) was estimated using a permutation procedure implemented in Genetix v.4.05. The IBD test was conducted both at the global scale, including all samples, and at a local scale considering the northern samples first (Figure 1), then central and southern samples (ICA, CON, ANG). We present both the IBD estimated with EPIC markers and those estimated using mitochondrial cytochrome *b* (cyt-b) sequence data from Durand et al. [32].

The program MIGRATE-n v3.4.4 [65] was used to infer the population size parameter Θ and the migration rate, *M* (*M* = *m*/μ, where *m* is the immigration rate per generation) among population clusters previously determined using TESS and/or BARRIER. MIGRATE-n v3.4.4 used the Brownian mutation model and mutation was considered to be constant for all loci. We used maximum likelihood (ML) implemented in MIGRATE-n to infer the various parameters and the 95% confidence intervals ( [66,67]). *F*_ST_ estimates were used as initial parameters for the estimation of Θ and *M*. For each locus, the ML was run for one hundred short and thirty long chains with 50,000 and 100,000-recorded genealogies, respectively, after discarding the first 10,000 genealogies (burn-in) for each chain. One of every 20 reconstructed genealogies was sampled for both the short and long chains. We used an adaptive heating scheme with 4 concurrent chains; the analyses were run on a cluster computer using one master and 6 compute nodes. Assuming a average mutation rate of 10^-5^ per locus per generation, average Θ estimates were translated to estimates of average effective population sizes (i.e. *N*_*e*_ = Θ/4μ) for each population cluster previously defined by TESS and/or BARRIER. Mutation rates of microsatellite loci are traditionally assumed to range from 10^-3^ to 10^-5^ per generation (e.g. [68,69]), but, at least in humans, microsatellite loci located within introns have been demonstrated to be less variable (i.e lowered mutation rate) than those present in intergenic regions [70]. We hence retained µ = 10^-5^ as a more appropriate mutation rate to analyze data in this study. Individuals genotyped for ≤5 loci were discarded from MIGRATE-n analyses. The value of Θ inferred with MIGRATE-n was used to compute the M-ratio test presented above. As credibility intervals derived for values of Θ inferred for each population cluster were found to broadly overlap, we used only one single value of Θ to compute the M-ratio test presented above (see ‘Results’ section).

## Results

### Genetic diversity

Allelic richness and observed levels of heterozygosity were quite variable among these loci, ranging from 5 alleles at the *CK6-2* and *Gapdh slow* loci to 17 alleles at locus *AldoB*. Concomitantly, observed gene diversity ranged from as low as 0.094 at locus *Gapdh slow* in MAU to 0.914 at locus *AldoB* in ANG (Table 2). Locus *Gapdh slow* presented high heterozygote deficits in nearly all samples analyzed with the exception of the ANG sample (Table 2). No significant deviation (after Bonferroni correction) from HWE was observed for the other loci (Table 2). Microchecker revealed that the recurrent deviation from Hardy-Weinberg equilibrium at locus *Gapdh slow* and, at a far lesser extent, at locus *CK6-3* (in one single sample, GUI, leading to departure from HWE; Table 2) may be potentially due to null alleles. Corrected allele frequencies following suggestions of Microchecker were considered hereafter for all analyses presented. Significant linkage disequilibrium was not found except, for one case (CAS: loci *AldoB-Myoglo2*).

**Table 2:** Diversity indices in *Ethmalosa* *fimbriata* estimated using 7 EPIC loci.

	MAU	SEN	SAL	GAM	CAS	GUI	ICA	CON	ANG
GAPDH fast									
*N*	60	54	41	47	59	54	48	53	28
*n*	10	12	11	11	11	12	11	12	11
*He*	0.713	0.811	0.842	0.833	0.835	0.871	0.732	0.854	0.802
*^f*	-0.006	-0.028	-0.073	-0.047	0.026	0.001	-0.025	0.028	-0.025
GAPDH slow									
*N*	52	50	27	23	53	53	49	52	31
*n*	4	4	4	5	3	5	5	4	4
*He*	0.094	0.679	0.324	0.491	0.254	0.479	0.656	0.609	0.235
*^f*	**0.388**	0.266	0.317	**0.651**	**0.631**	**0.412**	**0.660**	**0.339**	0.180
GAPDH slow*									
*N*	61	61	27	60	60	55	50	54	31
*n*	5	5	4	6	4	6	6	5	4
*He*	0.263	0.715	0.318	0.677	0.502	0.617	0.739	0.683	0.232
*^f*	0.073	-0.023	0.317	0.049	0.111	0.037	0.008	-0.022	0.180
AldoB									
*N*	59	60	43	44	60	54	50	53	25
*n*	12	11	11	13	11	12	13	16	12
*He*	0.857	0.865	0.867	0.872	0.858	0.837	0.835	0.886	0.914
*^f*	0.031	0.056	-0.073	0.009	-0.010	-0.085	-0.054	0.020	-0.051
CK6-1									
*N*	58	61	42	55	59	53	42	53	28
*n*	9	10	10	12	12	12	10	12	8
*He*	0.783	0.715	0.805	0.811	0.780	0.815	0.699	0.795	0.723
*^f*	-0.036	-0.009	0.084	-0.055	0.066	0.028	0.047	-0.069	0.112
CK6-2									
*N*	60	61	46	57	59	54	49	51	28
*n*	4	3	4	2	3	4	3	3	2
*He*	0.156	0.270	0.370	0.204	0.318	0.394	0.336	0.331	0.431
*^f*	0.042	0.089	0.119	0.054	0.256	-0.129	-0.095	-0.007	0.089
CK6-3									
*N*	52	61	40	54	60	54	50	54	21
*n*	5	6	3	4	6	5	6	5	5
*He*	0.550	0.574	0.573	0.558	0.644	0.546	0.547	0.562	0.556
*^f*	0.092	0.029	-0.003	0.072	0.122	0.255	0.123	0.079	0.147
Myoglo2									
*N*	54	59	40	39	52	50	50	51	29
*n*	5	5	5	4	4	5	6	6	5
*He*	0.702	0.634	0.628	0.591	0.656	0.618	0.596	0.629	0.687
*^f*	0.051	0.065	-0.076	0.047	0.004	-0.003	-0.074	-0.061	0.200

N: sample size, *n*: number of alleles, *He*: expected heterozygoty, ^ *f* estimate of Weir and Cockerham’s [50] equivalent of Wright’s [51] fixation index; average nuclear values across all seven nuclear loci. Bold values significant after Bonferroni correction. GAPDH slow*: index after allele frequency correction.

### Population structure

We observed significant and similar levels of differentiation across samples when using either uncorrected data set (*F*_ST_ = 0.032; *p* < 0.001) or the data set corrected for locus *Gapdh-slow* (*F*_ST_ = 0.031; *p* < 0.001). In contrast with the lack of population differentiation found within the northern biogeographic unit using mtDNA variability ( [32]), the application of EPIC markers, using the same samples, reveals that nearly all populations exploiting distinct estuaries are genetically differentiated (Table 3). Nevertheless, the samples SAL, GAM, CAS and GUI were not found to be differentiated when the correction for multiple tests was applied (Table 3).

**Table 3:** *Ethmalosa fimbriata* pairwise *F*_ST_-values among sampling localities at the cyt-b locus [32] (A), at seven nuclear DNA EPIC markers (B), and considering all loci (cyt-b + EPIC) (C).

		**North**						**Center**	**South**	
		MAU	SEN	SAL	GAM	CAS	GUI	ICA	CON	ANG
**A**	MAU	-	0.003	0.008	-0.006	-0.007	-0.008	**0.052**	**0.244**	**0.303**
	SEN		-	0.035	0.007	0.014	0.017	0.053	**0.251**	**0.308**
	SAL			-	-0.007	-0.003	-0.006	0.017	**0.205**	**0.261**
	GAM				-	-0.011	-0.009	0.034	**0.223**	**0.278**
	CAS					-	-0.008	0.035	**0.230**	**0.285**
	GUI						-	0.040	**0.246**	**0.301**
	ICA							-	**0.132**	**0.184**
	CON								-	0.022
	ANG									-
**B**	MAU	-	**0.061**	**0.031**	**0.042**	**0.026**	**0.045**	**0.097**	**0.053**	**0.058**
	SEN		-	**0.025**	**0.017**	**0.028**	**0.029**	**0.020**	**0.017**	**0.066**
	SAL			-	0.011	0.002	0.008	**0.048**	**0.022**	**0.035**
	GAM				-	0.001	**0.012**	**0.028**	**0.014**	**0.059**
	CAS					-	0.007	**0.044**	**0.020**	**0.039**
	GUI						-	**0.037**	0.013	**0.041**
	ICA							-	0.001	**0.072**
	CON								-	**0.035**
	ANG									-
**C**	MAU	-	**0.055**	**0.028**	**0.035**	**0.021**	**0.037**	**0.090**	**0.085**	**0.103**
	SEN		-	**0.031**	**0.019**	**0.029**	**0.029**	**0.034**	**0.067**	**0.118**
	SAL			-	0.008	0.002	0.006	**0.043**	**0.051**	**0.074**
	GAM				-	-0.001	0.009	**0.029**	**0.049**	**0.096**
	CAS					-	0.005	**0.042**	**0.055**	**0.082**
	GUI						-	**0.038**	**0.052**	**0.086**
	ICA							-	**0.029**	**0.090**
										
	CON								-	**0.034**
	ANG									-

Bold values significant after Bonferroni correction [58].

**Figure 2:**
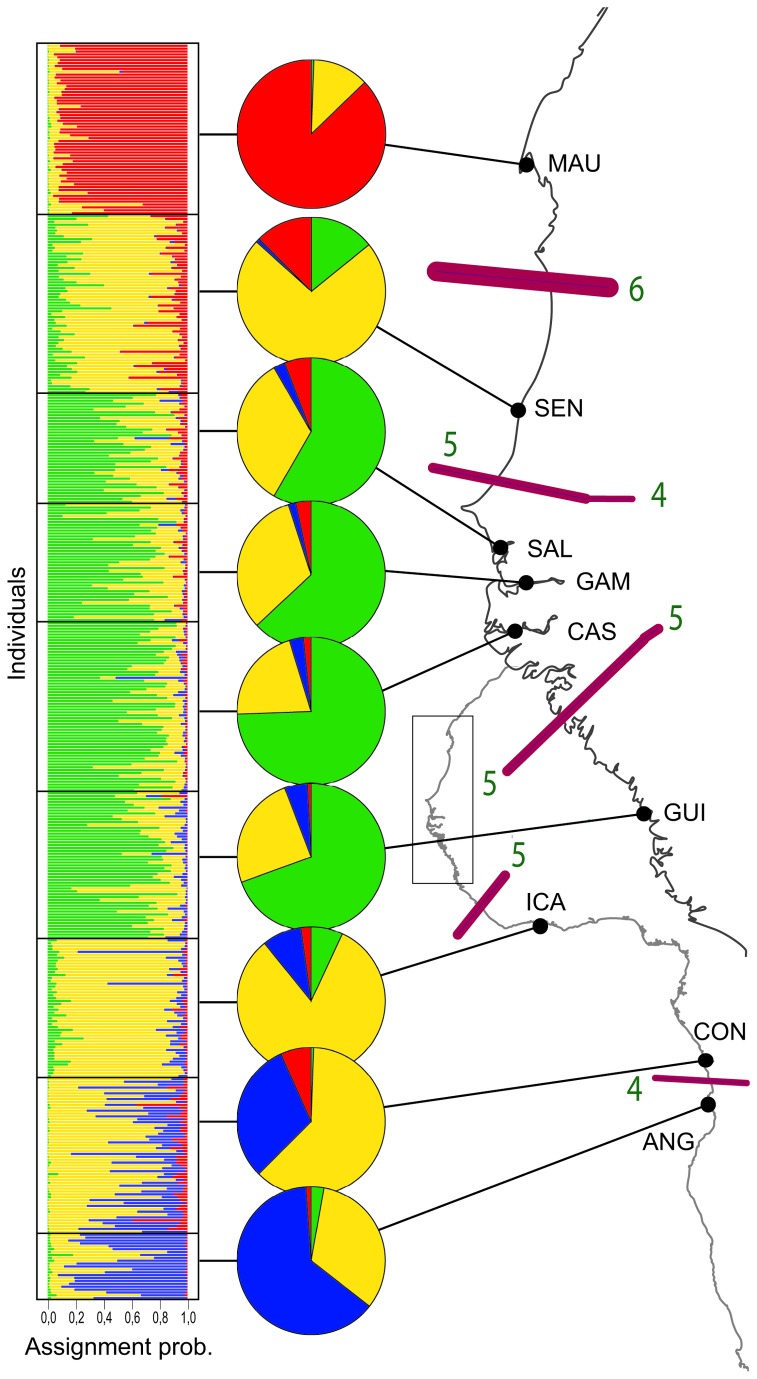
Genetic differentiation in Bonga shad along West African shoreline recovered with 7 EPIC loci. Admixture proportions at each sampling location (black dots) of the 4 genetic clusters recovered by TESS are shown in pies-charts. Gene flow barriers are highlighted using the spatial autocorrelation method, Barrier 2.2. are indicated by violet bars on the map, and their reliability was estimated using a bootstrap procedure on seven *F*_ST_ matrices (one per nuclear locus). Line thickness of gene flow barriers is proportional to the bootstrap value and green numbers indicate the number of loci that report significant genetic heterogeneity (i.e. number of loci supporting a genetic break).

Results from TESS supported the distinction of *K* = 4 distinct clusters of multilocus genotypes (Figure 2). Looking at the geographical distribution of clusters, the MAU and the ANG samples located at each limit of the species range demonstrated some genetic distinctiveness, each composed of a distinct dominant cluster (Figure 2). Contiguous samples SAL, GAM, CAS and GUI were found to group together (Figure 2), hence supporting the above-mentioned results based on estimates of F_ST_. The SEN sample located in the northern portion of the species range, and the ICA and CON samples located in the central-southern portion of the species range were found to be composed of individuals mostly belonging to the same dominant cluster, but with distinct proportions of alternative minor clusters (Figure 2).

BARRIER presented slightly different distinct zones of restricted gene flow than TESS, involving at least four loci (Figure 2). Congruent breaks are located (1) between the MAU and SEN samples at the upper northern distribution of species ranges (6 loci involved) (2), between the SEN and southern samples (5 loci) (3), between the GUI and ICA samples (5 loci), and (4) between the CON and ANG samples (4 loci). This last genetic break may be less apparent in terms of the number of loci supporting it because of a clinal trend between the dominant clusters inferred by TESS for the ICA, CON and ANG samples (Figure 2). BARRIER identified an additional break (5 loci) between the GUI and northern populations that was not found with TESS and not detected with *F*_ST_. The significance of this last barrier is thus questionable.

**Figure 3:**
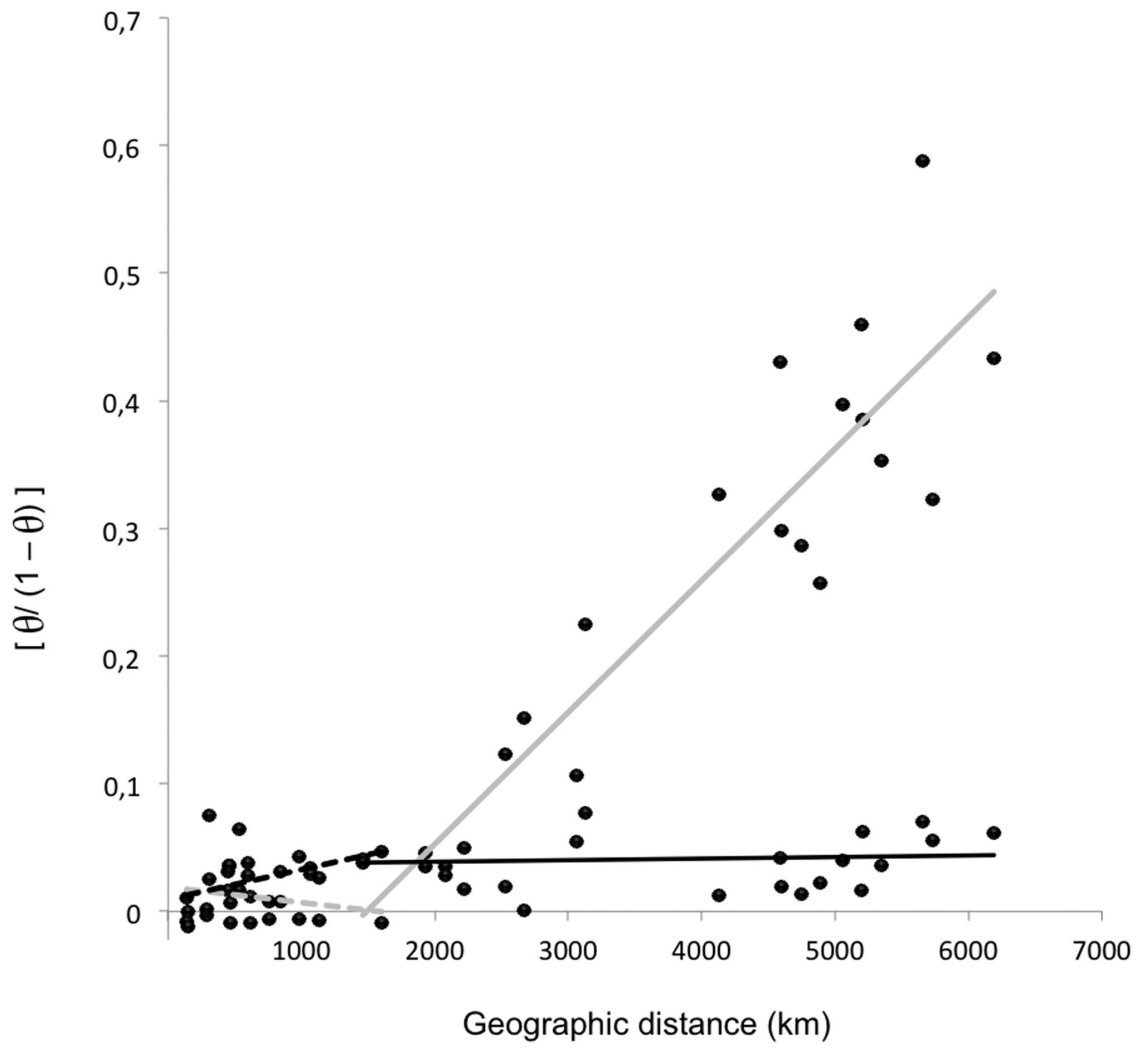
Relationship between the genetic [*F*_ST_/(1 -*F*_ST_); Rousset [64]] and the geographical distance (in km) among Bonga shad samples. Grey dashed line: regression within biogeographic groups (North = MAU+SEN+SAL+GAM+CAS+GIU, Center = ICA, South = CON+ANG) estimated using the mtDNA dataset [32] (R^2^=0.035); grey line: regression among biogeographic groups estimated with the mtDNA dataset (R^2^=0.818); black dashed line: regression within biogeographic groups estimated with the nuclear dataset (R^2^=0.266); black line: regression among biogeographic groups estimated with the nuclear dataset (R^2^=0.004).

Significant correlations were found for the EPIC markers when plotting [*F*_ST_ /(1 - *F*_ST_)] against geographical distance (*p* = 0.026; correlations remained significant when using ln[distance]; see Rousset [64]) for the northern mtDNA biogeographic unit previously defined by Durand et al. [32] (Figure 3). This relationship however does not hold true when considering the entire dataset spanning the entire African west coast. Interestingly, this situation is reversed when considering the mtDNA data set: no significant relationship exists within the northern biogeographic unit while a significant relationship exists when considering the entire dataset (Figure 3).

### Effective population size and migration

Values of Θ ranged from 0.860 and 0.961 for the population clusters located at the limits of species’ distribution range (i.e., the ANG and the MAU samples, respectively), and to 1.042 for the population cluster encompassing samples ranging from GUI to SAL (Table 4). Despite a slight decrease in estimates of Θ at distribution margins, 95% credibility intervals were found to overlap among the different clusters of Bonga shad (Table 4), except for the ANG population which was significantly lower than other estimates (*P* = 0.043). Assuming µ =10^-5^, these results translated to average values of *N*_*e*_ ranging from 2150 (95% CI: 1932.5-2582.5) for the southern ANG population of *E. fimbriata* to 2600 (95% CI: 2435-2827.5) individuals for the population cluster grouping samples from GUI to SAL. The inferred number of migrants between putative A and B clusters (i.e. *M*_A→B_^~^ *M*_B→A_) are reported in Table 5. Some caution is required in interpreting these results and trends in the number of exchanged migrants rather than their absolute numbers should be considered. The central cluster (SAL + GAM + CAS + GUI) was shown to produce more emigrants to the northern clusters (e.g. MAU) (*M*_[SAL+GAM+CAS+GUI]→MAU_ = 4.116) than the reciprocal estimate (*M*_MAU→[SAL+GAM+CAS+GUI]_ = 1.866), suggesting that the central population cluster might be the origin of the northern peripheral population as suggested by mtDNA [32]. A similar asymmetrical relationship was also observed between this central cluster and the southern clusters ([ICA+CON] and ANG; Table 5), also suggesting expansion.

**Table 4:** Estimates of the population size parameter (*Θ*) by MIGRATE-n [65] for each population cluster defined in Figure 2.

Cluster	Samples	*O* (± 95%CI)	M-ratio	*P* (M-ratio)*
A	MAU	0.961 (0.897-1.033)	0.721	0.140
B	SEN	1.026 (0.982-1.060)	0.763	0.337
C	SAL + GAM + CAS + GUI	1.042 (0.974-1.131)	0.783	0.402
D	ICA + CON	1.028 (0.960-1.089)	0.746	0.216
E	ANG	0.860 (0.773-0.962)	0.707	0.096

The M-ratio [55] determining the occurrence of a genetic bottleneck in each population cluster of *E. fimbriata* is also reported; together with the p-value associated with each observed M-ratio. * *p*-value inferred using *O* = 1, ∂ 3, and with 15% of mutations larger than single step mutations (see text for details).

**Table 5:** Maximum-likelihood estimates of inferred number of migrants (*M*_A→B_ and *M*_B→A_) by MIGRATE-n [65] between each pair of putative population clusters defined in Figure 2.

** - **	**B**	**MAU**	**SEN**	**SAL+GAM**	**ICA + CON**	**ANG**
**A**	-	-	-	**+CAS+GUI**	-	-
**MAU**		-	1.391	1.866	2.221	1.083
**SEN**		1.016	-	3.072	1.754	1.102
**SAL + GAM + CAS + GUI**		4.116	3.623	-	3.885	1.776
**ICA + CON**		2.116	1.905	2.256	-	2.180
**ANG**	-	0.960	0.938	0.510	0.614	-

Values of *M*_A→B_ are reported above the diagonal and values of *M*_B→A_ below the diagonal.

### Bottleneck

Using a common Θ of 1 as suggested by MIGRATE-n results (Table 4), no significant signatures of bottlenecks were found with the M*-*ratio test when samples were grouped according to population clusters previously identified (Table 4).

## Discussion

The aim of this study was to extend knowledge on the genetic population structure of the Bonga shad, an important resource for the Western African fishery. The finer resolution provided by the EPIC marker allows for greater precision in identifying the management units over an area where fishing pressure has dramatically increased over past decades. Until now, patterns of genetic differentiation in the Bonga shad were estimated using weakly polymorphic allozymes [27] and, subsequently, maternally-inherited mtDNA [32] that potentially did not reveal all components of the species population structure. We particularly focused on populations ranging from Mauritania to Guinea, an area where the Bonga shad is widely exploited and where ecological and physical features of the estuarine and coastal habitats are more likely to shape patterns of genetic variation. Indeed, we demonstrated that EPIC markers distinguished several, previously unknown, populations of the Bonga shad that are distinct from those previously defined. Fine scale genetic structuring is demonstrated in the northern part of the species’ distribution area, extending from Mauritania to Guinea.

### Nature of markers and large scale patterns of genetic differentiation

Using mtDNA, Durand et al. [32] demonstrated that three clades were present over the distribution area of the Bonga shad: a ‘northern group’ ranging from Mauritania to Guinea, a ‘central group’ covering most countries of the Gulf of Guinea, and a ‘southern group’ located from Gabon to Angola. Recent population expansion from a Pleistocene refuge located in the Gulf of Guinea towards more peripheral coastal habitats was also revealed [32]. The presence of a northern and a central group of the Bonga shad was also demonstrated by Gourène et al. [27] using allozymes. However, studies by Gourène et al. [27] and Durand et al. [32] were not fully congruent, as Gourène et al. [27] did not identify a southern group when using allozymes, despite also considering Congolese samples. Furthermore, the ‘northern groups’ defined by each group of authors were not identical. Based on mtDNA, Durand et al. [32] defined the ‘northern group’ as grouping all samples from Guinea to Mauritania, while Gourène et al. [27] found only one sample, located just north of Dakar, as genetically differentiated from others distributed over the remaining distribution range.

Results presented in this study, using EPIC, support the existence of several discrete populations. However, EPIC markers (i) have a substantially lower or higher genetic diversity than demonstrated for mtDNA and allozymes, respectively, (ii) did not show IBD among populations throughout the entire distribution range as demonstrated for mtDNA (i.e. comparing populations from the three distinct phylogeographical groups), and (iii) reported genetic differentiation at finer scales than found with mtDNA or allozymes. Indeed, within two of the three previously defined phylogeographic clades (the ‘northern group’ and the ‘southern group’), samples were found to be genetically distinct when using EPIC markers while they appeared genetically homogenous when using mtDNA. In the present study, the Congolese sample (CON) is genetically more similar to the Côte d’Ivoire sample (ICA) than to the Angolan sample (ANG), even though CON and ANG share most of their mtDNA haplotypes [32]. Despite the relatively low number of loci used in this study, the genetic distinctiveness of northern (MAU), central (SAL to GUI) and southern (ANG) samples is well illustrated by the high probability of membership of individuals within these populations to clusters defined by TESS.

The occurrence of weakly differentiated samples from SEN and ICA+CON that are geographically disjunct and belong to previously identified distinct mtDNA clades (see 32) is difficult to explain. It can either result from technical artefacts, such as homoplasy in some EPIC loci that can artificially link some populations, or reflect a truly higher genetic similarity among those remote populations, resulting from a complex dispersal history. Hence, EPIC markers appear quite useful to delineate local population structure, but estimates of population genetic differentiation may be biased downward for more divergent populations as the maximal value for differentiation is rapidly approached. For example, estimates of nuclear genetic differentiation between the samples located in the Banc d’Arguin (MAU) and the Senegal estuary (SEN) was *F*_ST_ = 0.061, equivalent to the level of genetic differentiation estimated for the two most distant populations analysed in this study and located more than 6000km apart (MAU and ANG; *F*_ST_ = 0.058). Considering all molecular markers simultaneously leads to the recognition of at least five to six distinct units of Bonga shad based on their nuclear and mtDNA backgrounds: ANG, CON, ICA, [GUI + CAS +GAM+SAL], SEN, and MAU.

### Isolation by distance and more complex scenarios

A major difference between mtDNA and EPIC marker data is the demonstration of IBD at distinct scales. At the larger scale considered in this study, mtDNA reflects an IBD pattern while the nuclear markers do not. A classical interpretation of this pattern is that mtDNA is more sensitive to changes in effective population size than nuclear DNA, resulting in reduced genetic drift at nuclear markers and slower nuclear differentiation. The differential susceptibility of markers to genetic drift has been documented for various marine fish species (e.g. [71], [72], [73]). However, at finer scales the situation is reversed, as IBD is found with nuclear markers among northern and among southern populations, while no IBD is found among mtDNA haplotypes within these regions. The relative strength of IBD may differ between markers and geographic regions depending on how far populations are from drift-migration equilibrium ( [74], [75]). According to theory, mtDNA reflects a situation of equilibrium between drift and gene flow in the Bonga shad, while nuclear DNA reflects a situation in which gene flow is prevalent at a local scale (IBD within clade) and drift is prevalent at a larger scale (no IBD among clades) [75]. For nuclear DNA, conditions encountered at each margin of the species’ distribution range certainly permit some level of localised dispersal between populations of finite size, resulting in IBD pattern between neighbouring populations at a more local scale ( [76], [75]). Castric and Bernatchez [77] documented a case similar to the pattern documented here. Using microsatellite loci, Castric and Bernatchez [77] showed that no detectable IBD was found in central coastal populations of the brook charr (*Salvelinus fontinalis*) in the Northwest Atlantic, while significant IBD was found among populations located at the northern and southern margins of the distributional range. This may have occurred because of a lower local effective population sizes in these areas compared to the central population. However, estimates of nuclear effective population sizes were not significantly different in samples distributed over the distribution range of the Bonga shad used in this study (*N*_*e*_^~^ 2500 ind. according to the mutation rate retained to derived values of *N*_*e*_: µ = 10^-5^). Reduction in *N*_*e*_ was not demonstrated using the M-ratio, indicating relative demographic population stability in the Bonga shad. The cause of observed IBD cannot be due only to differential susceptibility of nuclear and mtDNA markers to genetic drift and selection. Sex-biased dispersal and/or biased reproductive behaviour (i.e. partial homing) acting at different spatial or temporal scales could be alternative or complementary explanations to contrasted IBD patterns at nuclear and mtDNA markers. Biological data on sex-ratio, sex-specific dispersal and homing (degree of population closeness) are few and often dramatically distinct in the Bonga shad. For example, Blay and Eyeson [78] and N’Goran Ya [79] reported a female biased sex-ratio in the Gulf of Guinea (‘central population’), while Sheffer et al. [80] and Sheffers and Conand [81] reported equal representation of both sexes in populations belonging to the Senegal and Gambian waters (‘northern population’), respectively. Last, despite some reviews available on the biology of the Bonga shad [34], we do not know if this species illustrate the same anadromous life-cycle as other alosines. This strongly limits interpretation of observed patterns of IBD in the Bonga shad.

Furthermore, It should be noted that nuclear data may identify processes other than IBD, including secondary contact among populations of the Bonga shad. This is especially true for the transition between the central (ICA) and southernmost populations (CON, ANG); a sharper clinal pattern was found for mtDNA ( [32]) compared to the smoother transition documented for nuclear markers in this study. This may reflect differential introgression after secondary contact with the shape of the cline depending on the strength of selection, migration, and recombination that allows for introgression (e.g. [82]). Durand et al. [32] reported that the southern mtDNA clade has a complex history that may indeed reflect population expansion from the centre of the distribution range to the south. However, more complex scenarios leading to the reduction of gene flow, such as secondary contact, cannot not be ruled out. A better definition of the broad-scale landscape of gene flow sensu Marko and Hart [83] should motivate future studies on the evolutionary history of the Bonga shad.

### Genetic structure among northern populations: the interplay of the physical environment and alternative life cycles

In addition to the global patterns discussed above, results pertaining to the ‘northern group’ of the Bonga shad provide an assessment of factors that could have promoted nuclear genetic differentiation. In this area, TESS recognised four genetic clusters that globally matched results obtained with F_ST_-based estimates of genetic differentiation, with the exception of the Guinean (GUI) sample that was found genetically distinct from other populations with *F*_ST_, but not with TESS. Whatever the causes, results found in this study with EPIC markers differ from the perceived homogeneity revealed by mtDNA [32] and – with one exception (see above) – by allozymes [27]. Results hence provide evidence for lower genetic connectivity among northern coastal populations of the Bonga shad than previously thought.

Despite the elevated potential for population connectivity, little gene flow appears to connect populations distributed on each side of major barriers to dispersal identified herein. Results from BARRIER suggest that three barriers are relatively efficient in limiting gene flow: 1) the Cap Timiris trench in Mauritania, 2) the Kayar trench in Senegal, and 3) the Bijagos area (region between Guinea and Casamance) (Figure 1). Among these areas, only the Kayar trench has previously been proposed to impede fish dispersal ( [27,84]). These areas coincide with breaks in the distribution of Bonga shad adults along the coast identified by analyzing commercial captures and scientific sampling campaigns (IMROP and CRODT unpublished data; see Figure 1). It thus appears that shad dispersal is restricted by deep water as Bonga shad is restricted to shallow water (<10 m in Figure 1 or 20 m according [34]). In addition to physical barriers, the opportunity to complete alternative life cycles or to live in estuaries with distinct hydrological features may contribute to shaping patterns of genetic structure in the Bonga shad. Indeed, the MAU and SEN populations present distinct life cycles with the MAU population spending its whole life cycle in the marine environment, whereas the SEN population reproduces in the estuarine environment, which could represent the genetic isolation of distinct ecotypes. Interestingly, similar physical barriers and/or life cycle differences contributed to shape fine scale genetic structure in other estuarine species with a pelagic stage such as the rainbow smelt, *Osmerus mordax* or the American shad, *Alosa sapidissima* ( [85,86]; respectively, but nuclear and mtDNA data correlate in *smelt*, see 87). In American shad, Hasselman et al. [86] reported that physical barriers have a primary imprint on the genetic structure of this species, while characteristics of the reproductive cycle have a more subtle, secondary, influence. Nevertheless, ‘reproductive cycle’ has a different meaning depending on studies. In Hasselman et al. [86] ‘reproductive cycle’ describes the semelparous/iteroparous status of populations, while it expresses how the life cycle is completed in the Bonga shad (at sea vs estuary). Despite differences, such congruent patterns for species with similar life history traits underline the importance of environmental conditions and behaviour in the establishment of population structure. An interesting point of the present study is that the different hydrographical features of inland and coastal estuaries (i.e. no or large freshwater output to the sea, respectively) may also potentially impact genetic structure as individuals of the coastal GUI sample were found to be genetically distinct from individuals sampled in inland estuaries (SAL, GAM, CAS) with F_ST_-based estimates of genetic differentiation. As effective fishery management requires to correctly quantify connectivity patterns among stocks ( [88,89]), more detailed studies on the interplay of coastal physical barriers, hydrographical features of estuaries and life history patterns on the scale of dispersal of *E. fimbriata* are necessary to manage this resource more efficiently in the northern portion of its distribution range. This certainly needs to include both genetic and non-genetic markers such as otolith elemental microchemistry (e.g. [90], but see 91), or other techniques such as mass marking [92] and various modelling approaches (e.g. [93,94]).

## Conclusion

Although West African coastal waters are generally recognized as approaching overexploitation (e.g. [7,8]), few data exist describing population (‘stock’) structure for most species. Population genetic data offer an opportunity to describe such population structure (e.g. [19]), and the use of nuclear markers provided in this study has demonstrated that widely exploited marine species like the Bonga shad might be structured at a finer scale than previously recognized. Greater scrutiny of the exploitation of this species is called for and further assessment of population genetic structure of other West African fish species for which basic genetic knowledge is currently lacking is needed. In the context of the low resilience of most exploited fisheries [95] and faced with increased fishing pressure along the western coast of Africa ( [5,7]), such an evaluation is urgently needed to avoid the fate of many northern fisheries [96].

## Data Accessibility

Microsatellite data used in this manuscript: available on request.
